# Correction to “Multifactorial Induction of a “Scleroderma‐Like Pattern” Without Underlying Connective Tissue Disease: Diagnostic and Therapeutic Implications”

**DOI:** 10.1002/acr2.70028

**Published:** 2025-04-10

Angelo Nigro . Multifactorial Induction of a “Scleroderma‐Like Pattern” Without Underlying Connective Tissue Disease: Diagnostic and Therapeutic Implications. ACR Open Rheumatology. 2025;7: e11784.39739183 10.1002/acr2.11784PMC11685844

Correction for Panel B: revise the measurement of the apical diameter of the capillaries

Here is the corrected image of Panel B:

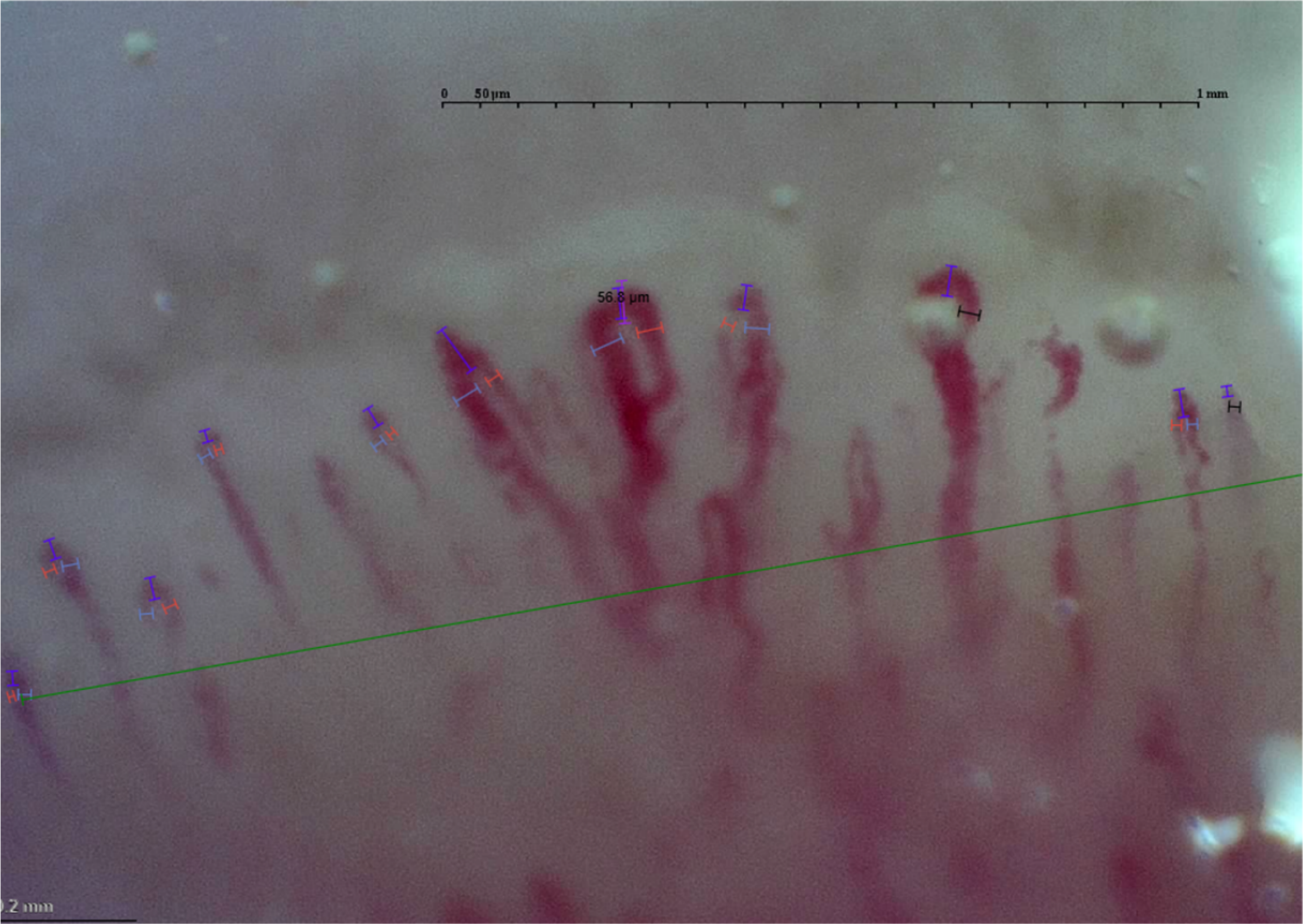

We apologize for this error.

